# Rapid Determination of Tetracyclines in Drinking and Environmental Waters Using Fully Automatic Solid-Phase Extraction with Ultra-Performance Liquid Chromatography–Tandem Mass Spectrometry

**DOI:** 10.3390/molecules29122921

**Published:** 2024-06-19

**Authors:** Tongtong Zhang, Xiangyang Zhang, Jiangmei Yu, Hongmei Hu, Pengfei He, Zhenhua Li, Yi Fang, Tiejun Li, Yuanming Guo

**Affiliations:** 1 Institute of Marine and Fisheries, Zhejiang Ocean University, Zhoushan 316021, China; 2 Key Laboratory of Sustainable Utilization of Technology Research for Fisheries Resources of Zhejiang Province, Zhejiang Marine Fisheries Research Institute, Zhoushan 316021, China; 3 Daishan County Science and Technology Innovation Center, Zhoushan 316200, China; 4 Zhoushan Ecological Environment Protection Technology Center, Zhoushan 316021, China

**Keywords:** tetracyclines, automated solid-phase extraction, isotope dilution, ultra-performance liquid chromatography–tandem mass spectrometry, water matrix

## Abstract

The abuse and irrational use of tetracyclines (TCs) in human medicine and animal husbandry has become a serious concern, affecting the ecological environment and human health. The aim of this study was to develop a sensitive and selective method using fully automatic solid-phase extraction coupled with ultra-performance liquid chromatography–tandem mass spectrometry for the determination of twelve TCs in water. Four isotope-labeled internal standards for TCs were used to correct matrix effects. Several parameters affecting extraction efficiency were systematically optimized, and the optimum experimental conditions found were 1.0 L water sample with 0.5 g/L Na_2_EDTA (pH 3.0) extracted and enriched by CNW HLB cartridge and eluted by 4 mL of acetone:methanol (*v*/*v*, 1:1). The enrichment factors were up to 798−1059 but only requiring about 60 min per six samples. Under the optimized conditions, the linearity of the method ranged from 0.2 to 100 μg/L for 12 TCs, the detection limits were as low as 0.01−0.15 ng/L, and the recoveries were in the range of 70%–118%, with relative standard deviations less than 15%. The developed method can be successfully utilized for the determination of 12 TCs in pure water, tap water, river water, and mariculture seawater. In summary, three and six TCs were detected in river water and mariculture seawater, respectively, with total concentrations of 0.074–0.520 ng/L (mean 0.248 ng/L) and 0.792–58.369 ng/L (12.629 ng/L), respectively. Tetracycline (TC) and oxytetracycline (OTC) were the dominant TCs in river water, while doxytetracycline (DXC) and OTC were dominant in mariculture seawater.

## 1. Introduction

Tetracyclines (TCs) are broad-spectrum antibiotics extensively used in human and veterinary medicine and agricultural feed additives [1]. They prevent bacterial growth by inhibiting protein biosynthesis [2,3] and exhibit activity against various Gram (+) and Gram (−) microorganisms, chlamydia, mycoplasmas, rickettsiae, and protozoan parasites [1]. Owing to their potent antibacterial properties and cost-effectiveness, TCs are very popular in Russia, China, the USA, the European Union, and South Korea, with approximately 23 kg of human daily consumption of tetracycline worldwide [4]. However, only a small fraction of TCs can be metabolized in the human and animal body, while 50–80% of TCs are excreted into the environment in the form of feces and excreta [1,5], posing potential threats to aquatic organisms and human health on a global scale. TCs are stable and difficult to oxidize in the environment but are unstable at extreme pHs, forming epi- and anhydro- products, and traditional wastewater treatment plants (WWTPs) cannot completely remove them, resulting in their continuous discharge into the environment, with residual concentrations ranging from ng/L to µg/L [5]. In order to protect human health, the European Union (EU), Codex Alimentarius Commission of the FAO/WHO, the US Food and Drug Administration (FDA), the Japanese Ministry of Health Welfare and Labor, and China have enacted maximum residue limits (MRLs) for the presence of TCs in foodstuffs of animal origin [6,7,8]. But until now, no MRLs have currently been set for TCs in environmental water. Thus, the development of high-precision, user-friendly, and prompt monitoring methods for the determination of TCs is crucial and essential to safeguard the environment, ensure the safety of food, and promote public health.

A wide range of analytical procedures have been developed for the determination of TCs in the environment and in organisms: enzyme-linked immunosorbent assays (ELISA), UV spectrophotometry (UV), capillary electrophoretic (CE), and high-performance liquid chromatographic methods (HPLC) coupled to detectors, such as fluorimetric detection (FLD), ultraviolet (UV), tandem mass spectrometers (MS/MS), and time-of-flight mass spectrometers (TOF MS) [6,9,10]. Currently, ultra-performance liquid chromatography–tandem mass spectrometry (UPLC–MS/MS) has been widely recommended due to its superior selectivity, sensitivity, and short analysis time [8]. Nevertheless, most previous studies mainly focused on several prototype TCs, such as tetracycline (TC), doxycycline (DXC), oxytetracycline (OTC), chlortetracycline (CTC), and lack of attention to their transformation products in water environment. TCs are known to have epimerization at the C-4 position and form 4-epitetracyclines due to hydrolysis and photolysis. Furthermore, 4-epimers and parent TCs show different in vitro antibiotic activities and toxicological effects [8]. It is necessary to take into consideration either tautomers and/or 4-epimers for the quantitative determination of TCs in water matrices.

Due to the varying matrices of environment water samples and low residue levels of target analytes, appropriate sample pretreatment techniques must be employed prior to instrumental analysis. To date, tremendous techniques have been developed to extract TCs in water matrices, such as solid-phase extraction (SPE) [11,12,13], dispersive solid-phase extraction (DSPE) [14], magnetic solid-phase extraction (MSPE) [15], and online SPE [16,17,18]. DSPE is economical, fast, and reproducible, but it suffers from poor sensitivity. MSPE has developed rapidly, but the preparation of magnetic adsorbents mainly depends on its stability, reproducibility, adsorption capacity, and selectivity. Traditional SPE works very well and is simple and reliable, but also has disadvantages such as a large sample volume, expensive cartridges, long sample pretreatment time, and multiple manual steps [19]. Recently, automatic online SPE coupled with the UPLC–MS/MS has been successfully applied in the determination of TC residues in environmental water, which dramatically reduced the sample volume (5–10 mL) and extraction time (13–20 min) [16,17]. However, the sensitivity, reliability, and repeatability need to be improved.

In this current study, a simple and rapid method was developed for the determination of 12 TCs in various water matrices using UPLC–MS/MS coupled with an automated cartridge-disk universal SPE system (in-built Poly-Sery HLB SPE cartridge). Matrix effects are an important issue in the development of the UPLC–MS/MS method, which affects the ionization efficiency of the target analytes [20,21]. Attempts were made to tackle matrix effects with four isotope-labeled internal standards (ILISs) for TCs. The optimized method was successfully applied to pure water, tap water, river water, and mariculture seawater, offering important evidence for the occurrence of tetracycline antibiotics in water environments.

## 2. Results and Discussion

### 2.1. Optimization of Automated Solid-Phase Extraction Procedures

The experimental parameters of the automated SPE, involving sorbent type, elution solvent and volume, chelating agent addition, and pH adjustment were conducted to achieve high extraction recovery. Ultrapure water spiked with 20 ng/L of TCs was used for optimization experiments, and all the experiments were performed in triplicate and the means of the results were used for optimization.

#### 2.1.1. Cartridge Sorbents

**Figure 1:**
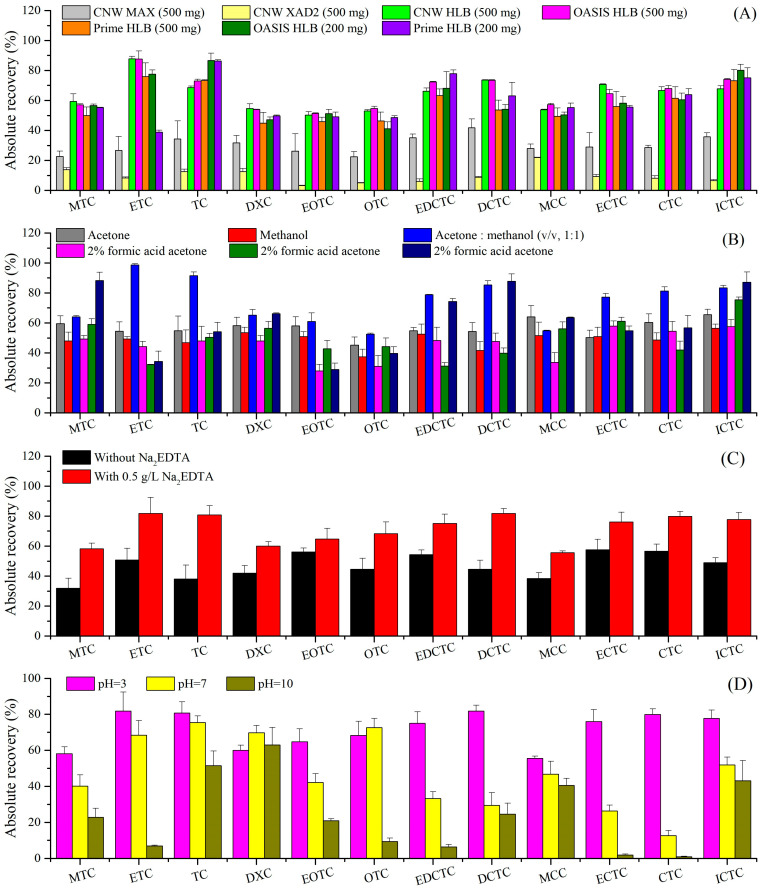
(**A**) Effect of cartridge sorbents (*n* =3); (**B**) effect of eluents (*n* =3); (**C**) effect of Na_2_EDTA addition (*n* =3); (**D**) effect of pH (*n* =3).

Adequate SPE cartridge choice was very important [9], and the amphoteric nature of TCs and the strong tendency to bind with silanol groups of silica makes the selection of cartridge sorbents challenging [10]. Nine different specifications or manufacturer commercial SPE cartridges (Table 1) were compared. CNW C18 (500 mg) and CNW MCX (500 mg) had the worst extraction effect, and 12 TCs were basically not detected. TCs can bind to silanol groups from C18, but the extraction recoveries were poor, perhaps due to difficulty in elution [9]. The extraction recovery was calculated by both the external standard method (absolute recovery) and the ILIS method (relative recovery). As shown in Figure 1A, among the other seven cartridges, the absolute recoveries of OASIS HLB (500 mg, range 51–88%, mean 66%) and CNW HLB (500 mg, 50–88%, 64%) were slightly higher than those of Prime HLB (500 mg, 45–76%, 58%) with OASIS HLB (200 mg, 41–87%, 61%), and Prime HLB (200 mg, 39–86%, 60%), but significantly higher than CNW XAD2 (500 mg, 3–22%, 10%) and CNW MAX (500 mg, 22–42%, 30%). Overall, HLB cartridges are preferred over other cartridges, which is consistent with the previous literature reports [10].

Meanwhile, the relative recoveries of OASIS HLB (500 mg, 78–126%, 96%) and CNW HLB (500 mg, 70–122%, 95%) for all analytes were satisfactory after internal standard correction (Supplementary Materials, Figure S1). Nevertheless, the relative recoveries of other cartridges for MTC, ETC, EOTC, EDCTC, and ECTC were less than 70%. Additionally, the price of CNW HLB (500 mg) is twice as cheap as OASIS HLB (500 mg) [21]. Hence, CNW HLB (500 mg) was chosen for further study.

#### 2.1.2. Elution Solvent and Eluent Volume

Due to the high polarity of TCs, eluents with large polarity are commonly used for elution [9]. Six eluents with 8 mL were employed to evaluate their influence on recovery. As shown in Figure 1B, acetone:methanol (*v*/*v*, 1:1) exhibited the best elution performance, with absolute recoveries ranging from 53% to 99% (mean 74%), followed by 2% oxalate methanol (range 29–88%, mean 62%) and acetone (45–65%, 57%). The lowest absolute recoveries were obtained when using 2% formic acid methanol (31–75%, 49%), methanol (37–56%, 49%), and 2% formic acid acetone (28–58%, 46%). However, except for 2% oxalate methanol with low recovery for ETC (69%), it is worth noting that the relative recoveries of 12 TCs ranged from 70% to 110% for the other five eluents (SI, Figure S1). Thus, acetone:methanol (*v*/*v*, 1:1) was chosen as the optimal elution solvent.

After the elution solvent was fixed, the effect of the eluent volume was also explored by varying the volume from 4 to 16 mL. As shown in SI, Figure S2, the absolute recoveries increased slightly when the eluent volume increased from 4 mL (range 56–82%, mean 72%) to 8 mL (53–99%, 74%) and continued to increase the volume; the extraction efficiency remained basically unchanged. In addition, the relative recoveries of 12 TCs ranged from 72% to 110%, irrespective of the eluent volume used. Therefore, to shorten the nitrogen blowing time, 4 mL acetone:methanol (1:1, *v*/*v*) was used for elution in subsequent experiments.

#### 2.1.3. Effect of Na_2_EDTA

Due to the presence of two ketone groups on positions 1 and 11 (SI, Figure S3), TCs possess a strong tendency to form chelate complexes with metal ions [22], which affects the extraction. Therefore, Na_2_EDTA was usually added for competitive metal chelation in a previous study [14]. Meanwhile, the excessive Na_2_EDTA enhanced the matrix effect. As shown in Figure 1C, the absolute recoveries with 0.5 g/L Na_2_EDTA addition (range 56–82%, mean 72%) were higher than those without Na_2_EDTA addition (32–58%, 47%). In addition, the relative recoveries were in the range of 70–106% regardless of whether Na_2_EDTA was added (SI, Figure S1), which met the analytical method requirements.

#### 2.1.4. Effect of pH Value

TCs are amphoteric compounds, such that their ionization forms are influenced by solution pH. Thus, TCs may cause protonation or deprotonation and behave as cationic (pH < 3.3), zwitterion (3.3 < pH < 9.0), or anionic forms (pH > 9.0) based on three distinct pK_a_ values (Table 2) [23]. In this study, the effect of pH on the extraction was investigated within the range of pH 3–10. The conditions of pH were regulated by using hydrochloric acid and sodium hydroxide. Figure 1D shows the absolute recoveries at pH 3 (range 56–82%, mean 72%) were higher than those at pH 7 (13–75%, 48%), and significantly higher than those at pH 10 (1–63%, 24%). Furthermore, the relative recoveries for some TCs (eg MTC) were higher than 120% at pH 7 and pH 10 (SI, Figure S1). The results were basically consistent with the previous studies in which pH 3–5 is preferred [24]. Thus, the sample pH was fixed at 3.0 in this study.

### 2.2. Matrix Effect

**Figure 2:**
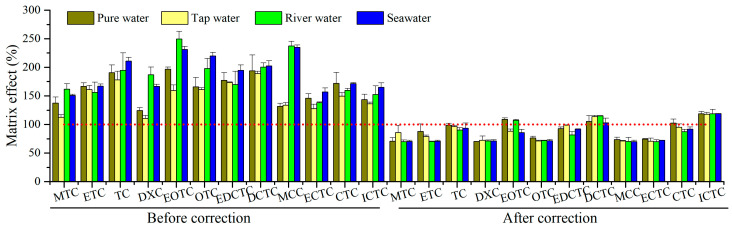
Matrix effects in four different water matrices.

A major drawback of UPLC–MS/MS has been recognized to be matrix effects triggered by co-elution substances, which may lead to either signal suppression or enhancement of target analytes [25]. In this study, the matrix effects (ME) were estimated following our previous work [26]. The calculation formula was ME(%) = (A_e_ − A_0_)/A_s_ × 100%, where A_e_, A_0_, and A_s_ were the signal intensity of the spiked extracts, unspiked extracts, and standard solution, respectively. ME values corresponding to 100% indicate that there are no matrix effects, while ME values >100% or <100% denote signal enhanced or suppressed. As shown in Figure 2, signal enhanced was observed for target TCs in four test matrices (ME range 110–250%). The results are consistent with matrix effects for TCs found in groundwater and surface water samples from sites around the United States [27]. Satisfactorily, the matrix effects were significantly minimized after ILIS calibration, and ME values were in the range of 70–119%. Otherwise, it is necessary to adopt more stringent matrix purification methods to reduce matrix effects, or establish the matrix-matched calibrations for TC quantitation [28]. Thereafter, we chose to use the internal standard method for quantitative analysis.

### 2.3. Evaluation of the Method Performance

Under optimum conditions, the performance of the proposed automated SPE–UPLC–MS/MS method was evaluated regarding its linearity, sensitivity (limits of detection (LODs) and limits of quantitation (LOQs)), enrichment factors (EFs), accuracy, and precision (Table 3). The calibration curves of 12 TCs were established with ILISs in concentrations of 0.2–100 μg/L. Good linear relationships were observed, with the correlation coefficient (r^2^) ranging from 0.9959 to 0.9996. The LODs and LOQs were in the range of 0.01–0.15 ng/L (S/N = 3) and 0.03–0.50 ng/L (S/N = 10), respectively. EFs were determined by calculating the ratio of the equilibrium concentration of analytes in the initial mobile phase to the original concentration of analytes in the aqueous phase, with values of 798−1059 in this study. Intra- (*n* = 5) and inter-day (*n* = 5) precisions were calculated by extracting the analytes from ultrapure water samples at the level of 20 ng/L, and relative standard deviations (RSDs) lower than 12% and 14% were obtained, respectively (Table 3). These results demonstrated a high sensitivity and excellent repeatability of the proposed method.

Furthermore, the accuracy of the method was evaluated by three different spiked concentrations of 12 TCs (2, 20, 100 ng/L) (SI, Table S1). It can be seen that in tap water, river water, and seawater, the recoveries of the TCs ranged from 70 to 114%, 72 to 118%, and 72 to 115%, respectively, with RSDs of 0.9–7.0%, 0.3–12.1%, 0.8–13%, and 0.3%–14.1% (*n* = 5), respectively. As a result, the recovery and precision of this method were satisfactory, which could meet the requirements for the determination of TCs in real environmental water.

The comparison of the present method with published methods is given in SI, Table S2. It can be seen that the LODs of the present method are comparable to manual SPE–UPLC–MS/MS [11,12] but superior to those obtained with online SPE–LC–MS/MS [18], online SPE–UPLC–MS/MS [16,17], direct injection (DI) UPLC–MS/MS [29], DSPE–UPLC–MS/MS [14], reciprocating magnetic-field-assisted (RMF) SPE–LC–MS/MS [23], and manual SPE–UPLC-FLD [13], and they are much lower than MSPE–UPLC–MS/MS [15] and vortex-assisted (VA) fatty acid-based ternary deep eutectic solvent (TDES) spectrophotometer [30]. Moreover, the proposed method exhibited good precision and recoveries for more kinds of TCs, and a comparable or shorter pretreatment time. The proposed method showed significant promise due to its high sensitivity and effectiveness for rapid analysis of trace TCs in a water environment.

### 2.4. Real Water Analysis

**Figure 3:**
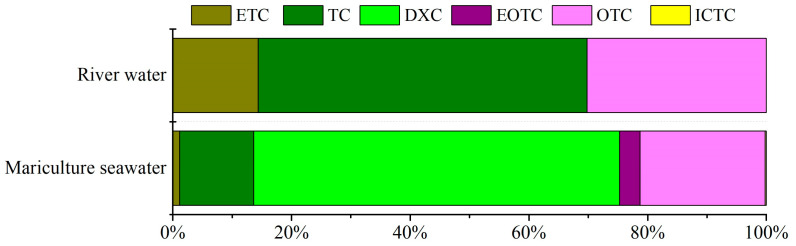
Distribution of TCs in river water and mariculture seawater.

The proposed automated SPE–UPLC–MS/MS method was applied for the determination of 12 TCs in Wahaha pure water, tap water, river water, and mariculture seawater to examine its feasibility. TCs were not detected in Wahaha pure water and tap water. However, three and six TCs were detected in river water and mariculture seawater, respectively, with the total concentration of TCs (∑TCs) ranging from 0.074 to 0.520 ng/L (mean 0.248 ng/L) and 0.792 to 58.369 ng/L (12.629 ng/L), respectively (SI, Table S3). As shown in Figure 3, the river water is dominated by TC (55%) and OTC (30%), while the mariculture seawater is dominated by DXC (62%) and OTC (21%). The results are consistent with many previous studies [1,12,31]. For example, among the target compounds, TC, OTC, and DXC were the dominant antibiotics detected in the drinking water sources of the lower Yangtze River [12]. The concentrations of TCs in mariculture seawater were significantly higher than those in river water. The maximum concentrations of TC, OTC, and DXC were found in mariculture seawater at 8.170, 10.857, and 44.186 ng/L, respectively, because of the wide use of TCs as growth promoters in numerous aquaculture farms [1,31]. More attention should be paid to the occurrence of TCs in the aquaculture environment.

## 3. Materials and Methods

### 3.1. Chemicals and Reagents

Twelve TC standards, i.e., methacycline (MTC), 4-epitetracycline (ETC), tetracycline (TC), doxycycline (DXC), 4-epioxytetracycline (EOTC), oxytetracycline (OTC), 4-epidemeclocycline (EDCTC), demeclocycline (DCTC), meclocycline (MCC), 4-epichlortetracycline (ECTC), chlortetracycline (CTC), and isochlortetracycline (ICTC) were purchased from ANPEL Laboratory Technologies (Shanghai, China). They had four corresponding ILISs: TC-D_6_, DXC-D_3_, and CTC-^13^C-D_3_ were obtained from Toronto Research Chemicals (Toronto, Canada), and OTC-^13^C_22_^15^N_2_ was purchased from Shanghai ZZBIO Co, Ltd. (Shanghai, China). The stock standard solutions of 12 TCs (1 mg/L) and four ILISs (1 mg/L) were prepared in methanol and stored in the dark at −20 °C. Then, fresh calibration standard solutions were prepared daily by diluting the mixed standard solution with the initial mobile phase.

HPLC-grade methanol and acetone were provided by Merck (Darmstadt, Germany), and formic acid, oxalic acid, and ethylenediaminetetraacetic acid disodium salt (Na_2_EDTA) were obtained from Sigma-Aldrich (St. Louis, MO, USA). Ultrapure water (18.2 MΩ/cm) was prepared with a Milli-Q Plus 185 system (Millipore Corporation). Nine SPE cartridges were investigated in this study for sample extraction, including 500 mg CNW C18, 500 mg CNW MCX, 500 mg CNW MAX, 500 mg CNW XAD2, 500 mg CNW HLB from CNW Technologies (Duesseldorf, Germany), 500 mg OASIS HLB, 200 mg OASIS HLB, 500 mg Prime HLB, and 200 mg Prime HLB from Waters (Milford, MA, USA).

### 3.2. Sampling and Preparation

A total of six Wahaha pure water samples, six tap water samples, six river water samples, and six mariculture seawater samples were collected from shop stores, laboratories, the urban river in Zhoushan, and marine aquaculture farms surrounding Zhoushan and Taizhou, East China, respectively, in April 2024, and the sampling locations are shown in Figure S4. The collected river water or mariculture water samples were filtered through 0.45 μm glass fiber filters to remove insoluble impurities and then stored at 4 °C until extraction.

### 3.3. Automated Solid-Phase Extraction

The fully automatic SPE method was used to extract target TCs from water samples. The extraction procedure was performed with an automated cartridge-disk universal SPE system (LabTech, China) (SI, Figure S5), which can process six samples simultaneously. The extraction efficiencies using nine commercial SPE cartridges were evaluated. The nature and properties of these cartridges are given in Table 1.

The optimized extraction procedure is as follows: the SPE cartridges were preconditioned with 8 mL of methanol and 8 mL of acidified ultrapure water (pH 3.0). Then, 1.0 L of the filtered water sample with 0.5 g Na_2_EDTA, spiked with 20 ng of four mixed ILISs, and adjusted to pH 3.0 was passed through the preconditioned cartridge, followed by 15 mL of ultrapure water and 5 mL of 5% methanol. After sample loading and rinsing, the SPE cartridges were dried under N_2_-blowdown for 10 min, and eluted with 4 mL of acetone:methanol (*v*/*v*, 1:1). Finally, the collected eluents were concentrated to dryness with a stream of nitrogen at 40 °C (45 position N-EVAP/13165 nitrogen Evaporator, Organomation, Berlin, MA, USA), redissolved in 1 mL of the initial mobile phase, and filtered through 0.22 μm filter. Then, 5 μL of this solution was injected into the UPLC–MS/MS system for analysis.

### 3.4. Instrumental Analysis

A Waters Acquity UPLC I-Class system (Waters, Milford, MA, USA) coupled with a Xevo TQ-S triple quadrupole mass spectrometer (Waters, Manchester, UK) in multiple reaction monitoring (MRM) modes was used to analyze the target TCs. Post-injection of 5 μL of the sample extracts, the analyses were separated on a Waters BEH C18 column (2.1 mm × 100 mm, 1.7 μm) at a flow rate of 0.30 mL/min, and the column temperature was maintained at 40 °C. Gradient elution was performed with 0.1% formic acid in ultrapure water (eluent A) and methanol (eluent B) starting with 90% A (0−2 min), 78% A (3 min), 65% A (7 min), 15% A (9 min), 5% A (12 min), and finally, 90% A (12.1−15 min).

**Figure 4:**
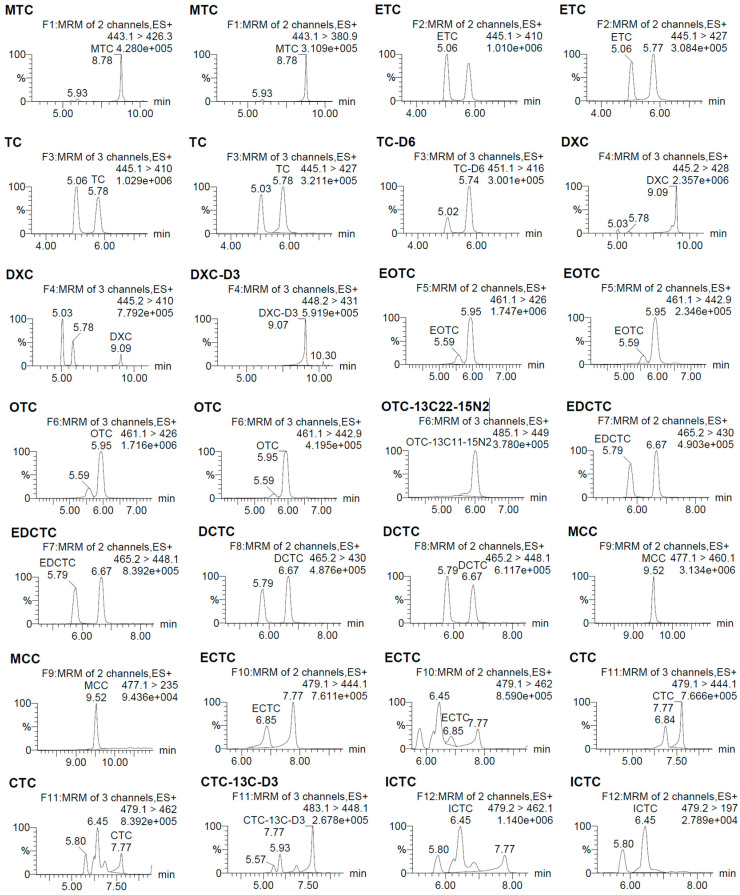
UPLC–MS/MS chromatograms of TCs standard.

The MS/MS was operated in positive electrospray ionization (ESI+), with operating conditions as follows: capillary voltage at 3.0 kV, desolvation temperature at 500 °C, source temperature at 150 °C, desolvation gas flow at 1000 L/h, and cone gas flow at 150 L/h. Nitrogen (99.99%) was used as the desolvation and cone gas, and argon (99.9999%) as the collision gas. Table 2 shows the MRM transition parameters for each compound, and their mass spectrums are shown in Figure 4.

## 4. Conclusions

A sensitive and reliable method was developed for trace determination of 12 TCs in various water matrices using automated SPE and UPLC–MS/MS. The isotope-labeled internal standard method was used to compensate for matrix effects on quantitation. The validation test of the proposed method showed excellent results in terms of calibration linearity, method detection limit, precision, and recovery. Three and six TCs were detected in river water and mariculture seawater, respectively. TC and OTC were the dominant TCs detected in river water, while DXC and OTC were the main components in mariculture seawater. The developed method provides a routine tool to obtain accurate occurrence data of TCs in drinking and environmental waters that will permit risk assessment associated with exposure to these contaminants at low-nanogram to nanogram-per-liter levels.

## Figures and Tables

**Table 1:** Nature and properties of tested SPE cartridges.

SPE Cartridge	Dimensions	Sorbent Properties
CNW LC-C18	40–63 μm ^a^, 500 mg ^b^, 6 mL ^c^	C18 alkyl-bonded silica
CNW MCX	92 μm, 500 mg, 6 mL	mixed-mode cation exchange
CNW MAX	35–45 μm, 500 mg, 6 mL	mixed-mode anion exchange
CNW XAD2	694 μm, 500 mg, 6 mL	non-ionic reticulated styrene–divinylbenzene polymer
CNW HLB	66 μm, 500 mg, 6 mL	Hydrophilic–lipophilic-balanced reverse phase polymer
OASIS HLB	58.4 μm, 500 mg, 6 mL	Hydrophilic–lipophilic-balanced reverse phase polymer
Prime HLB	60–80 μm, 500 mg, 6 mL	Hydrophilic–lipophilic-balanced reverse phase polymer
OASIS HLB	58.4 μm, 200 mg, 6 mL	Hydrophilic–lipophilic-balanced reverse phase polymer
Prime HLB	60–80 μm, 200 mg, 6 mL	Hydrophilic–lipophilic-balanced reverse phase polymer

^a^ Particle size; ^b^ Sorbent bed weight; ^c^ SPE tube volume.

**Table 2:** The physicochemical properties and MS/MS conditions of 12 TCs.

Abbreviation	Full Name	Molecular Weight	Solubility (mg/L)	Log *K*_OW_	pKa	Retention Time (min)	Precursor Ion (*m*/*z*)	Product Ion (*m*/*z*)	Cone Voltage (V)	Collision Energy (eV)
MTC	Methacycline	442.42	7550	−1.37	3.50, 7.60, 9.20	8.78	443.1	380.9, 426.3 *	34	20, 25
ETC	4-Epitetracycline	444.44	3880	−1.33	4.50, 11.02	5.06	445.1	410.0 *, 427.0	25	20, 14
TC	Tetracycline	444.43	231	−1.30	3.30, 7.68, 9.69	5.78	445.1	410.0 *, 427.0	25	20, 14
TC-D_6_		450.47				5.74	451.1	416.0 *	25	20
DXC	Doxycycline	444.44	630	−0.02	3.02, 7.97, 9.15	9.09	445.2	410.0, 428.0 *	30	24, 18
DXC-D_3_		447.48				9.07	448.2	431.0 *	30	18
EOTC	4-Epioxytetracycline	460.43	501	−1.50	NA	5.59	461.1	426.0 *, 442.9	20	20, 15
OTC	Oxytetracycline	460.44	313	−1.12	3.27, 7.32, 9.11	5.95	461.1	426.0 *, 442.9	20	20, 15
OTC−^13^C_22_^15^N_2_		484.44				5.95	485.1	449.0 *	20	20
EDCTC	4-Epidemeclocycline	464.85	NA	−0.81	7.46	5.79	465.2	430 *, 448.1	25	22, 17
DCTC	Demeclocycline	464.85	1520	−1.14	8.23	6.67	465.2	430.0 *, 448.1	30	22, 18
MCC	Meclocycline	476.86	NA	NA	NA	9.52	477.1	235.0, 460.1 *	30	20, 17
ECTC	4-Epichlortetracycline	478.88	NA	−0.53	6.90	6.85	479.1	444.1 *, 462.0	15	20, 18
CTC	Chlortetracycline	478.88	630	−0.62	3.30, 7.55, 9.15	7.77	479.1	444.1 *, 462.0	15	20, 18
CTC-^13^C-D_3_		482.88				7.77	483.1	448.1 *	15	20
ICTC	Isochlortetracycline	478.88	NA	1.99	4.50, 11.02	6.45	479.1	197.0, 462.1 *	30	25, 18

NA: not available; * quantitative ion.

**Table 3:** Analytical characteristics of the proposed method.

Analyte	ILIS	Linear Range (μg/L)	Regression Equation	*r* ^2^	LOD ^a^ (ng/L)	LOQ ^b^ (ng/L)	EFs	Precision, RSD (%, *n* = 5)	
								Intra-Day	Inter-Day
MTC	OTC-^13^C_22_^15^N_2_	0.2−100	y = 0.19x + 0.02	0.9979	0.10	0.30	828	4.81	5.05
ETC	TC-D_6_	0.2−100	y = 0.69x − 0.0.02	0.9978	0.02	0.06	798	4.82	4.39
TC	TC-D_6_	0.2−100	y = 0.42x + 0.05	0.9992	0.02	0.06	978	10.58	8.85
DXC	DXC-D_3_	0.2−100	y = 0.97x + 0.19	0.9996	0.01	0.03	934	1.62	3.28
EOTC	OTC-^13^C_22_^15^N_2_	0.2−100	y = 0.43x + 0.07	0.9980	0.03	0.10	865	11.25	12.51
OTC	OTC-^13^C_22_^15^N_2_	0.2−100	y = 0.80x − 0.03	0.9991	0.03	0.10	907	10.43	13.51
EDCTC	CTC-^13^C-D_3_	0.2−100	y = 0.27x + 0.02	0.9959	0.15	0.50	826	11.86	10.16
DCTC	CTC-^13^C-D_3_	0.2−100	y = 0.25x + 0.02	0.9974	0.15	0.50	1059	7.43	11.63
MCC	OTC-^13^C_22_^15^N_2_	0.2−100	y = 0.59x + 0.13	0.9984	0.06	0.20	886	4.55	5.04
ECTC	CTC-^13^C-D_3_	0.2−100	y = 0.36x − 0.04	0.9986	0.01	0.03	903	10.99	9.34
CTC	CTC-^13^C-D_3_	0.2−100	y = 0.50x + 0.53	0.9990	0.01	0.03	880	3.82	3.93
ICTC	CTC-^13^C-D_3_	0.2−100	y = 1.15x + 0.30	0.9982	0.03	0.10	962	11.68	13.49

^a^ LOD (S/N = 3); ^b^ LOQ (S/N = 10).

## Data Availability

The original contributions presented in the study are included in the article/Supplementary Material, further inquiries can be directed to the corresponding author/s.

## Supplementary Materials

The following supporting information can be downloaded at: https://www.mdpi.com/article/10.3390/molecules29122921/s1, Figure S1: (A) Effect of cartridge sorbents (n = 3); (B) Effect of eluents (n = 3); (C) Effect of Na2EDTA addition (n = 3); (D) Effect of pH (n = 3); Figure S2: Effect of eluent volume on extraction efficiency: 1.0 L of ultrapure water (containing 0.5 g/LNa_2_EDTA, pH 3.0) spiked with 20 ng/L TCs (n = 3); Figure S3: Chemical structures of the 12 TCs; Figure S4: Map of sampling sites in study area, East China; Figure S5: Automatic cartridge-disk universal solid phase extraction system (LabTech, China); Table S1: Recoveries of real water samples spiked with TCs obtained by applying the proposed automated SPE UPLC-MS/MS method; Table S2: Comparison of different methods for the analysis of TCs in water; Table S3: Concentrations (ng/L) of TCs in river water and mariculture seawater samples (ng/L).
