# Supplementary material for: Rapid Determination of Tetracyclines in Drinking and Environmental Waters Using Fully Automatic Solid-Phase Extraction with Ultra-Performance Liquid Chromatography–Tandem Mass Spectrometry
Source: Molecules. 2024 Jun 19;29(12):2921. doi: 10.3390/molecules29122921 (PMC11206360; doi:10.3390/molecules29122921)
Supplement: Supplementary file 1 [file molecules-29-02921-s001.zip › molecules-3065591-supplementary.pdf]

SUPPORTING INFORMATION

Rapid Determination of Tetracyclines in Drinking and Environmental Waters using Fully Automatic Solid-Phase Extraction with Ultra-Performance Liquid Chromatography–Tandem Mass Spectrometry

Tongtong Zhang 1,2,†, Xiangyang Zhang 3,†, Jiangmei Yu 4, Hongmei Hu 2,*, Pengfei He 2, Zhenhua Li 2, Yi Fang 2, Tiejun Li 2,* and Yuanming Guo 2

1 Institute of Marine and Fisheries, Zhejiang Ocean University, Zhoushan 316021, China

2 Key Laboratory of Sustainable Utilization of Technology Research for Fisheries Resources of Zhejiang Province, Zhejiang Marine Fisheries Research Institute, Zhoushan 316021, China

3 Daishan County Science and Technology Innovation Center, Zhoushan 316200, China

4 Zhoushan Ecological Environment Protection Technology Center, Zhoushan 316021, China

* Correspondence: huhm@zju.edu.cn (H.H.); litiejun1982@126.com (T.L.);

Tel.: +86-580-2299883 (H.H.); +86-580-2299886 (T.L.); Fax: +86-580-2299881 (H.H.); +86-580-2299881 (T.L.)

† These authors contribute equally to this work.

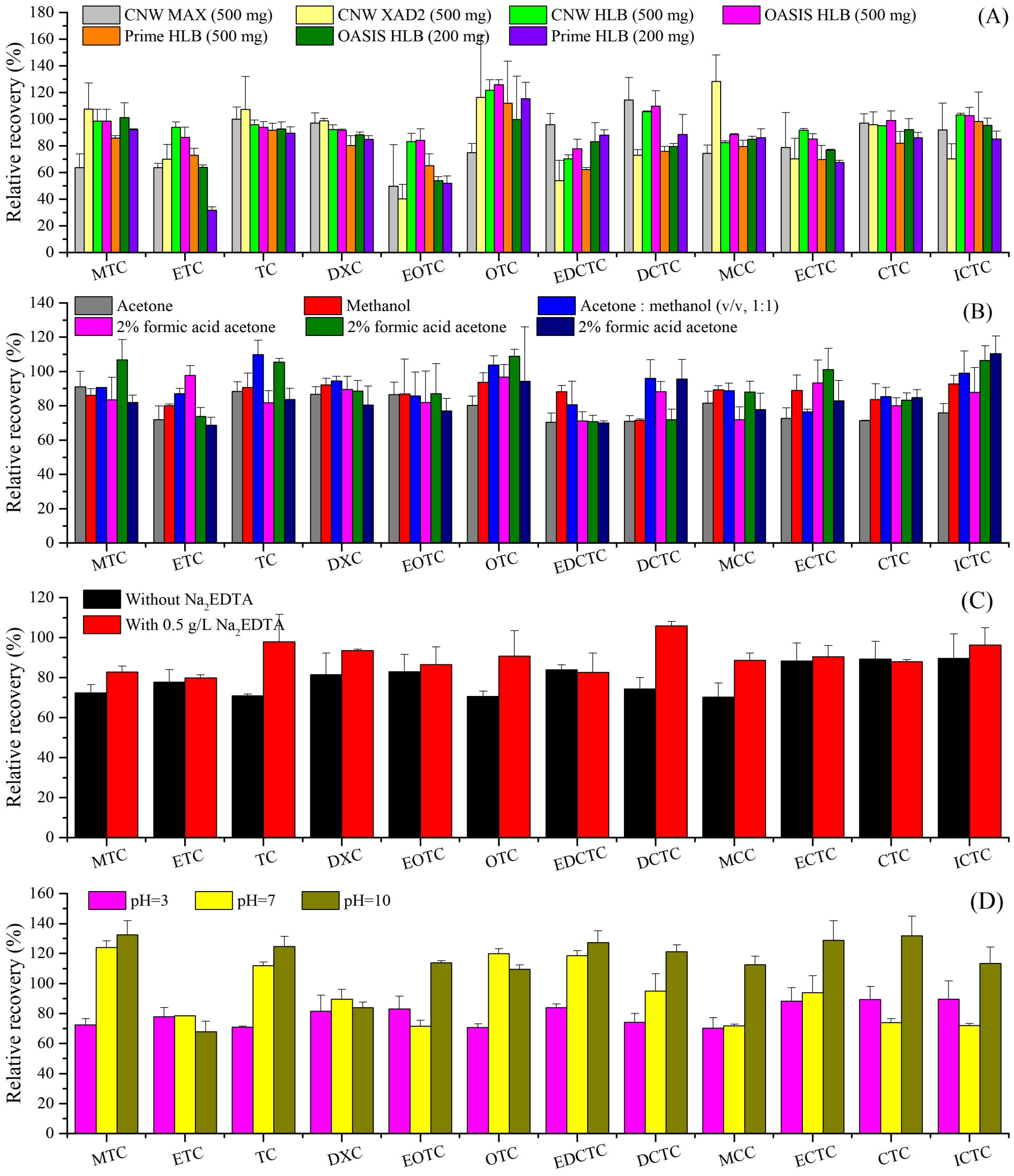

Figure S1. (A) Effect of cartridge sorbents (n=3); (B) Effect of eluents (n=3); (C) Effect of Na_2EDTA addition (n=3); (D) Effect of pH (n=3).

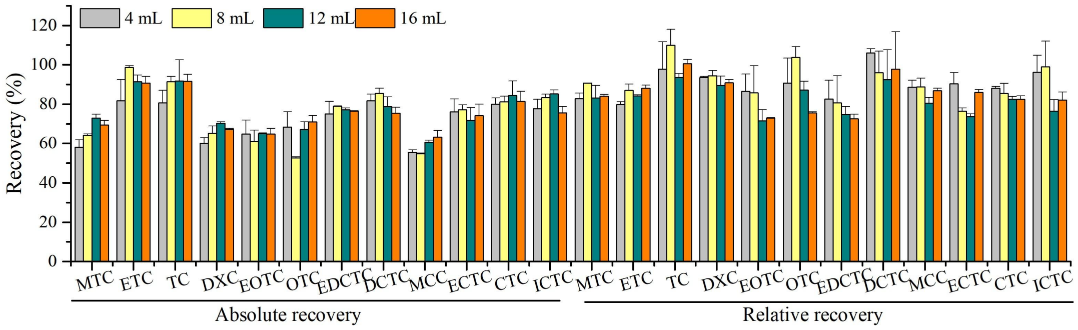

Figure S2. Effect of eluent volume on extraction efficiency: 1.0 L of ultrapure water (containing 0.5 g/LNa_2EDTA, pH 3.0) spiked with 20 ng/L TCs (n=3).

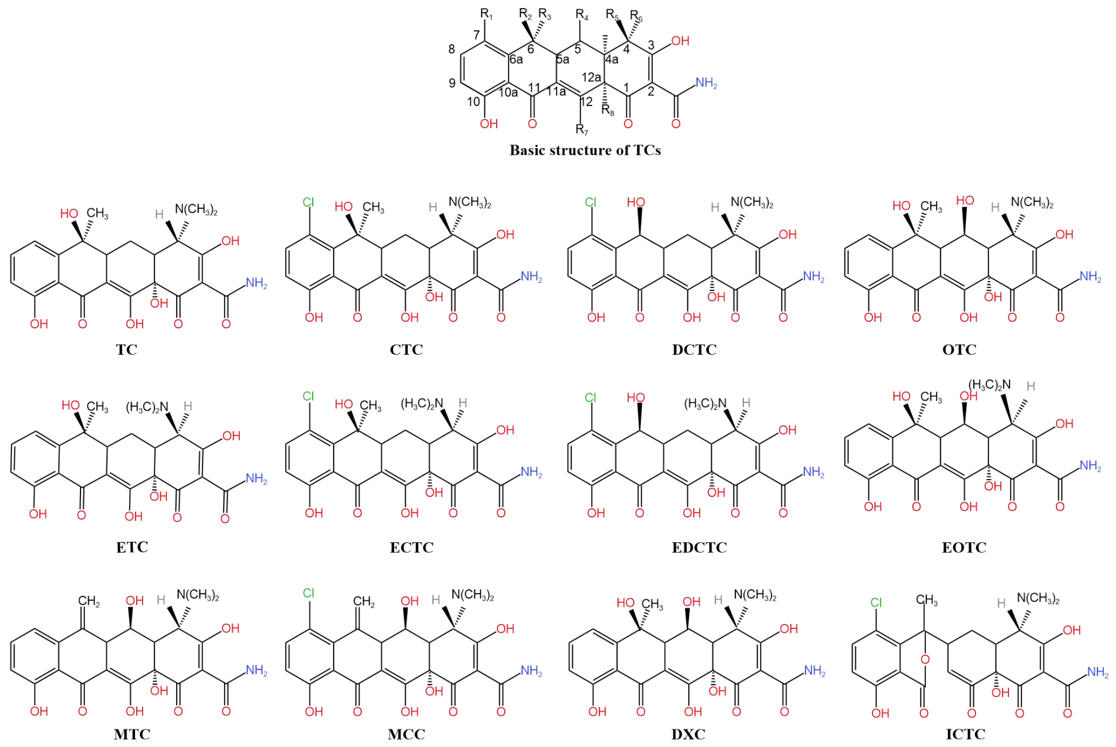

Figure S3.   Chemical structures of the 12 TCs.

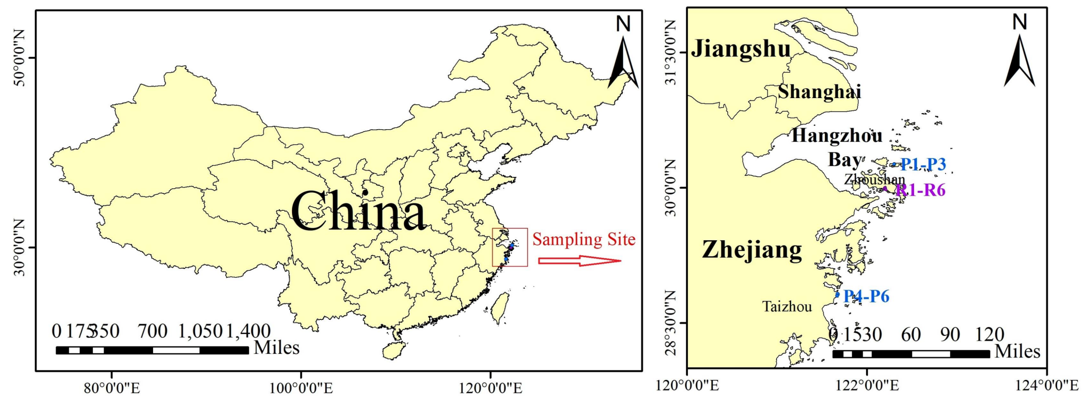

Figure S4. Map of sampling sites in study area, East China.

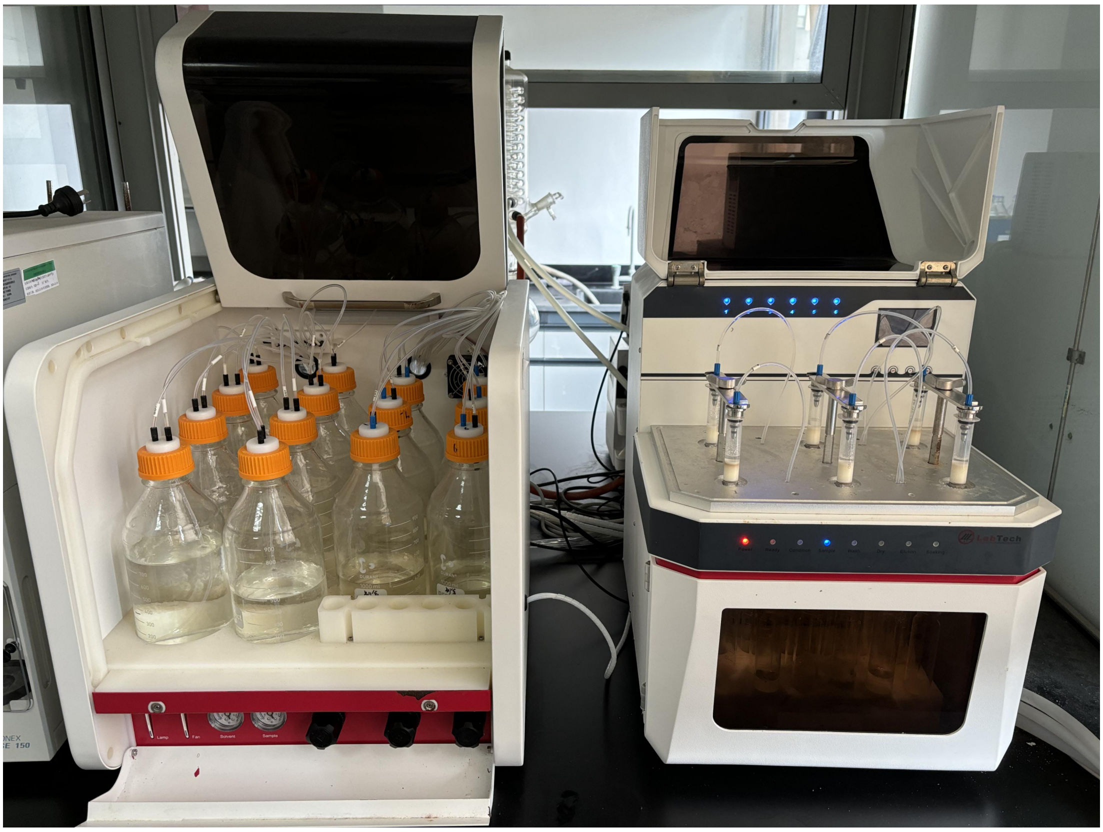

Figure S5. Automatic cartridge-disk universal solid phase extraction system (LabTech, China).

Table S1. Recoveries of real water samples spiked with TCs obtained by applying the proposed automated SPE UPLC-MS/MS method.

Analyte	Tap water (n=5)						River water (n=5)						Seawater (n=5)					
	Spiked: 2 ng/L		Spiked: 20 ng/L		Spiked:100 ng/L		Spiked: 2 ng/L		Spiked: 20 ng/L		Spiked: 100 ng/L		Spiked: 2 ng/L		Spiked: 20 ng/L		Spiked: 100 ng/L	
	Recovery (%)	RSD (%)	Recovery (%)	RSD (%)	Recovery (%)	RSD (%)	Recovery (%)	RSD (%)	Recovery (%)	RSD (%)	Recovery (%)	RSD (%)	Recovery (%)	RSD (%)	Recovery (%)	RSD (%)	Recovery (%)	RSD (%)
MTC	95	4.5	87	3.3	81	4.2	106	9.6	85	4.7	72	2.1	82	14.1	91	12.5	79	5.7
ETC	81	9.6	89	0.6	106	1.9	106	4.1	106	3.6	108	0.8	115	4.7	114	4.3	104	3.3
TC	99	7.6	92	6.5	96	5.1	107	3.6	110	1.2	108	9.7	106	0.3	95	6.2	96	1.8
DXC	94	1.2	94	0.7	88	2.8	84	12.1	95	7.2	96	7.8	108	7.1	93	4.4	85	3.1
EOTC	89	8.3	97	6.3	70	5.6	84	10.8	118	2.2	111	8.3	115	2.8	114	2.8	114	7.1
OTC	114	2.8	99	1.5	100	7.2	76	4.8	118	1.2	111	1.1	109	4.8	115	1.3	114	5.9
EDCTC	73	0.9	90	1.2	74	6.3	75	4.7	85	11.0	102	4.8	79	11.1	77	12.1	74	6.5
DCTC	91	6.9	90	12.1	97	11.5	93	5.1	90	5.1	107	13.0	105	10.9	74	7.3	80	2.9
MCC	87	8.7	89	4.0	93	7.9	104	11.8	97	7.8	86	10.8	111	2.2	73	5.1	72	3.7
ECTC	92	1.4	88	9.3	101	10.8	112	7.8	117	3.8	115	1.2	113	8.7	113	7.6	113	3.2
CTC	88	12.1	93	9.8	111	0.3	107	5.1	109	3.3	105	1.8	110	11.6	94	7.6	101	4.6
ICTC	108	5.6	99	4.3	76	6.4	74	6.0	91	12.7	91	8.6	111	6.1	114	7.2	106	12.4

Table S2. Comparison of different methods for the analysis of TCs in water.

Method	N*	Sample volume	Sample pretreatment	sorbent	Elution or disperser solvent	Processing time (min)	LOD (ng/L)	Recovery (%)	RSD (%)	Ref.
UPLC-MS/MS	1	2 mL	Ditect injection (DI)	/	/	~10 min	21.4	81–94	<14	[29]
manual SPE UPLC-MS/MS	4	1 L	Addition Na_2EDTA, pH adjustment of 2.0	200 mg of Oasis HLB	10 mL of 2% formic acid solution in methanol/acetonitrile(4:1, v/v)	> 250 min	0.018–0.108	81–96	<10	[11]
manual SPE UPLC-MS/MS	11	2 L	Addition Na_2EDTA, pH adjustment of 3.0	500 mg of Oasis HLB	12 mL of methanol	> 250 min	0.016–1.65	84–107	<10	[12]
On-line SPE LC-MS/MS	3	900 µL	pH adjustment of 4.0	Agilent Zorbax 80 SB-C8 column	Methanol (solvent C): water acidified to pH 4 with orthophosphoric acid (solvent D) 5:95 (v/v) (C/D)	13 min	1.2–1.5	83–93	<10	[18]
On-line SPE UPLC-MS/MS	4	5 mL	/	Oasis HLB	water containing 1% formic acid (A), methanol containing 0.1% formic acid (B) and methanol/acetone/n-hexane (1:1:1) (C)	13 min	0.5–1.5	82–103	<6	[17]
On-line SPE UPLC-MS/MS	5	10 mL	Addition formic acid	HyperSep Retain PEP[a]	methanol/acetonitrile(1:1, v/v)	20 min	1.32–7.91	92–123	<22	[16]
manual SPE LC-FLD	4	250 mL	Addition Na_2EDTA, pH adjustment of 3.4	30 mg of Oasis HLB	1 mL of methanol containing 1% trifluoroacetic acid (TFA)	> 250 min	40–150	>80	<10	[13]
DSPE UPLC-MS/MS	4	1 mL	3 g/L Na_2EDTA	40 mg primary secondary amine sorbent	/	~10 min	20–30	68–91	<20	[14]
MSPE HPLC-MS/MS	3	10 mL	pH adjustment of 4.0	20 mg Magnetic activated carbon	2 mL of acetone	~30 min	100–950	91–102	<10	[15]
RMF-SPE-LC-MS/MS	6	200 mL	Addition Na_2EDTA	10 mg of carboxyl-modified magnetic nanoparticles (CMNPs)	100 µL mixed eluent of methanol, acetonitrile, and 0.02 mol/L oxalic acidsolution (10:20:70, v/v)	/	12–74	95–111	<12	[23]
VA TDES Spectrophotometer	1	5 mL	pH adjustment of 5.0 Addition NaCl	/	600 µL of fatty acid-based ternary deep eutectic solvent (TDES)-3, 350 µL of acetonitrile	~10 min	1000	94–99	<6	[30]
automated SPE UPLC-MS/MS	12	1 L	Addition Na_2EDTA, pH adjustment of 3.0	500 mg of CNW Poly-Sery HLB	4 mL of methanol : acetone (v/v, 1:1)	~60 min	0.01–0.15	70–118	<15	Present work

* Number of TCs evaluated.

Table S3. Concentrations (ng/L) of TCs in river water and mariculture seawater samples (ng/L).

		ETC	TC	DXC	EOTC	OTC	ICTC	∑TCs
River water (n=6)	Mean	0.036	0.137	ND	ND	0.075	ND	0.248
	Min	ND	ND	ND	ND	ND	ND	0.074
	Max	0.094	0.425	ND	ND	0.233	ND	0.520
	DF(%)	33	67	0	0	67	0	100
Mariculture seawater (n=6)	Mean	0.144	1.576	7.786	0.438	2.658	0.027	12.629
	Min	ND	ND	ND	ND	ND	ND	0.792
	Max	0.457	8.170	44.186	2.208	10.857	0.086	58.369
	DF(%)	67	83	50	50	83	17	100

DF, detection frequency.

ND, not detected.
